# *Vitex Rotundifolia* Fractions Induced Apoptosis in Human Breast Cancer T-47D Cell Line via Activation of Extrinsic and Intrinsic Pathway

**DOI:** 10.31557/APJCP.2019.20.12.3555

**Published:** 2019

**Authors:** Gul-e-Saba Chaudhry, Rehmat Jan, Muhammad Naveed Zafar, Habsah Mohammad, Tengku Sifzizul Tengku Muhammad

**Affiliations:** 1 *Institute of Marine Biotechnology, Universiti Malaysia Terengganu, Kuala Terengganu, Malaysia, *; 2 *Department of Chemistry, Quaid-i-Azam University, Islamabad, Pakistan. *

**Keywords:** Vitex rotundifolia, cytotoxicity, apoptosis, annexin V/PI, T-47D

## Abstract

**Objective:**

Breast cancer is the most frequently diagnosed cancer worldwide. The main objective of the present study was to evaluate the cytotoxic effects and mechanism of cell death induced by the extract and fractions of Vitex rotundifolia (leaves) in breast cancer cell line, T-47D.

**Methods:**

The cytotoxicity activity was measured using MTS assay. The mode of cell death was analysed by early (phosphatidylserine externalization) and late apoptosis (DNA fragmentation). The caspases 8, 9, 3/7 and apoptotic proteins bax, bcl-2 study were done by western blot and ELISA method.

**Results:**

The methanol extract was found to inhibit 50% growth of T-47D cells at the concentration of 79.43µg/ml respectively after 72hr. From seven fractions, fraction F1, F2 and F3 produced cytotoxicity effects in T-47D cell line with IC_50_ (72hr) < 30µg/ml. The results obtained by Annexin V/PI apoptosis detection assay and TUNEL assay suggest that active fractions of Vitex rotundifolia induced early and late apoptosis (DNA fragmentation) in T-47D cell line. Moreover, western blot analysis and Caspase Glo^TM^ luminescent assay demonstrated that fractions F2 and F3 triggered apoptotic cell death via activation of caspases -8, -9 and -3/7 and up-regulation of Bax and down-regulation of Bcl-2 protein. Furthermore, chemical profiling confirms the presence of potential metabolites (vitexicarpin) in fractions of Vitex rotundifolia.

**Conclusion:**

Thus, the present study suggests the remarkable potential of active metabolites in fractions of Vitex rotundifolia as future cancer therapeutic agent for the treatment of breast cancer.

## Introduction

Cancer is one of major cause of death in developed and developing countries (Nithya et al., 2014). Remarkable progress has been made in oncology and various strategies for the treatment of cancer have been developed. However, despite the progress, cancer continues to affect patients and is the leading cause of death worldwide as no solution medication has been developed to cure the disease, especially when the cancer cells have metastasized (Mou et al., 2011). 

Breast cancer is the most frequently diagnosed cancer and is common cause of death in women both in developed and developing countries. Different therapies are used to block breast cancer progression, however in developing countries, survival rates for breast cancer are still poor and it accounts for 22.9% of all cancers in women (Guerrero et al., 2017; Mohankumar et al., 2013). Current therapies for cancer such as chemotherapy, radiotherapy or immunotherapy primarily act by causing cell death in cancer cells. Therefore, inability to exert their antitumor effect by activating cell death pathways (apoptosis) in cancer cells may lead to resistance of cancer to these treatment approaches. In addition, some undesired side effects are often linked to occur as a result of chemotherapy (Desai et al., 2008). 

There is plethora of evidence that suggest natural products such as the plant-derived products have long been utilized for the treatment of a variety of ailments including cancer. Natural compounds derived from a variety of medicinal plants led to the development of anticancer drugs with reduced adverse side effects (Desai et al., 2008). The studies conducted to date reveal that various potential biologically active compounds such as iridoids, flavonoids, diterpenoids, derivatives, and phytosteroids are present in species of genus Vitex, which possess analgesic, anti-inflammatory, anti-cancer, antimicrobial, antioxidant, hepatoprotective, antihistamine, and antiasthmatic properties. (Meena et al., 2011). *Vitex agnus-castus, Vitex negundo, Vitex trifolia *and *Vitex rotundifolia*, have been studied for their pharmaceutical properties and biological activities (Rani and Sharma, 2013). 

*Vitex rotundifolia* (Beach Vitex) is a deciduous, sprawling shrub that is widely distributed in coastal areas of Japan, Southeast Asia, Pacific Islands, and Australia (Ohtsuki et al., 2014). Dried ripened fruit of *Vitex rotundifolia* has been used as a traditional medicine to treat headaches, migraines, eye pain, asthma, chronic bronchitis, night blindness, gastrointestinal infections and inflammation for many years as a folk medicine (Kim et al., 2017). Cancer cell death caused by different plant derived compounds involves the mechanism of apoptosis (Solowey et al., 2014; Greenwell et al., 2015). Furthermore, *Vitex rotundifolia* possess anti-cancer (Ko et al., 2000; Zhou et al., 2013; Song et al., 2017; Ko et al., 2001; Song et al., 2017; Song et al., 2018), antioxidant (Yoshiokaa et al., 2004; Kim, 2009; Rani and Sharma, 2013) and antibacterial properties (Kawazoe et al., 2001; Bae et al., 1998).

Therefore, the present study was carried out to investigate the cytotoxic effects of the extract and fractions prepared from leaves of *Vitex rotundifolia* in breast cancer cell lines, MCF-7 and T-47D. In addition, the mode of cell death was also discussed focusing on early and late apoptosis and mechanism of cell death induced by the active fractions on breast cancer cell lines was also studied. 

## Materials and Methods

*Materials*

T-47D cell line was purchased from American Type Cell Culture, USA. Roswell Park Memorial Institute (RPMI) 1640 medium, Penicillin-streptomycin solution, Fetal Bovine Serum (FBS) and other cell culture supplies were obtained from Sigma Aldrich, USA. CellTiter 96^TM^ AQueous Non-Radioactive Cell Proliferation Assay kit (MTS), Annexin V-FITC Apoptosis Detection Kit DeadEnd™ Fluorometric Apoptosis Detection Kit (TUNEL) and Caspases-Glo^TM^ kit -3/7, 8 and 9 were purchased from Promega, USA. Mouse monoclonal primary antibodies and anti-mouse m-IgGk BP-HRP secondary antibody used in the study were obtained from Santa Cruz Biotechnology, Inc. Europe. Vitexicarpin was purchased from Permula, USA. All other chemical solvents were purchased from Thermo Fischer Scientific, UK. 

*Preparation of extract and fractions*

*Vitex rotundifolia* leaves were collected from Teluk Ketapang, GPS cordinates (5º22’54 N.103º06’59 E) Kuala Terengganu, Malaysia. The specimens were deposited in the Institute of Marine Biotechnology, UMT, Malaysia. The leaves were freeze dried and ground to powdered form by using mechanical grinder. The crude methanol extract was prepared by using cold extraction technique. Furthermore, seven fractions, hexane (F1), hexane: dichloromethane (F2), dichloromethane (F3), dichloromethane: ethyl acetate (F4), ethyl acetate (F5), ethyl acetate: methanol (F6) and methanol (F7) were prepared from the methanol extract using solid phase extraction (SPE) technique. 

*MTS assay*

CellTiter 96^TM^ AQueous Non-Radioactive Cell Proliferation Assay (MTS assay) was used to evaluate the cytotoxicity activity of extract and fractions of Vitex rotundfolia on T-47D cell line (Mohammad, TST et al., 2017). Briefly, T-47D cells were cultured at a cell density of 6×10^3^ cells/ well and incubated with seven different concentrations of extract and fractions for 24hr, 48hr and 72hr. Vincristine sulphate was used as positive control in MTS cell proliferation assay and untreated negative control cells were cultured with complete medium containing 1% (v/v) DMSO. After that, CellTiter 96^TM^AQueous Solution Reagent (20μl) was added into each well for 3hr at 37°C. Finally, the absorbance was recorded using ELISA 96-well plate reader at 490nm 

*Annexin-V/ PI staining assay*

*Annexin V-FITC Apoptosis Detection Kit was used to study the early and late apoptosis*

T-47D cells were treated with active fractions and vincristine sulphate (positive control) at concentration of their respective IC_50_ (72hr) and incubated for 12hr, 24hr and 36hr. Negative control cells were cultured with complete medium containing 1% (v/v) DMSO. Annexin V/PI staining was performed as done previosuly (Gul-e-Saba et al., 2018; Zafar et al., 2019). Cell apoptosis was observed under ImageXpress Micro XLS Widefiled High Content Screening (HCS) (Sunnyvale, USA).

*TUNEL assay*

DeadEnd^™^ Flourometric Apoptosis Detection System (TUNEL assay) was used to study DNA fragmentation induced apoptosis in T-47D cells. The cells were cultured in labtek chamber slides at a cell density of 2×10^4^ cells/chamber and incubated at 37ºC in a humidified atmosphere in the presence of 5% (v/v) CO_2_ for 24hr. Next, the old medium was discarded and replaced with a fresh medium containing the active fractions at a concentration of IC_50_ (72hr) and incubated for 36hr. The method was performed as previousy reported (Gul-e-Saba et al., 2019). After treatment, the cells was fixed by using 4% paraformaldehyde solution in PBS (pH 7.4) for 25 mins at 4°C. The fixed cells were then permeabilized by immersing the slides in 0.2% (v/v) Triton X-100 solution (prepared in PBS). The images were observed under ImageXpress Micro XLS Widefiled High Content Screening (HCS) (Sunnyvale, USA) to detect the green fluorescence of FITC-labeled apoptotic cells. 

*Western blot analysis*

Western blot analysis was performed to elucidate the mechanism of the apoptosis induced by the active fractions of *Vitex rotundifolia*, and activity of caspase and Bcl-2 family proteins was examined as previously reported method (Gul-e-Saba et al., 2019). Cell lysate was prepared by using Cytoplasmic Extraction Reagents and protein concentrations were calculated using BSA Protein Assay Kit (Bio-Rad Laboratories, USA). Subsequently, 150μg of proteins were separated on a 10% SDS- poly- acrylamide gel electrophoresis and electro-transfer to PVDF membrane. The membrane was then blocked in a blocking buffer for 2 hours and incubated with mouse monoclonal primary antibodies overnight at 4°C. Next, the membrane was incubated with anti-mouse m-IgGk BP-HRP secondary antibody for 2hr. Gel doc 2000 and GS-800 calibrated densitometer (BioRad, USA) were used for imaging and analysis of detected proteins. 

*Caspase assay*

The levels of active caspases -8, -9 and -3/7 were determined by using Caspase Glo™ Assay (Promega, USA). Breast cancer cell line T-47D was treated with active fractions of *Vitex rotundifolia* at a concentration of IC_50_ (72hr) and incubated for ten different time points (1hr, 3hr, 6hr, 9hr, 12hr, 16hr, 20hr, 24hr, 36hr and 48hr) at 37ºC in a humidified atmosphere in the presence of 5% (v/v) CO_2_. The method was done according to manufacturer’s protocol. The luminescence of each sample was measured and recorded using luminometer. 

*Thin layer chromatography*

TLC analysis was performed on TLC silica gel 60 F_254_ plates to identify the presence of standard compound Vitexicarpin (casticin) in the active fractions of *Vitex rotundifolia*. The fractions and standard compound were diluted in a requisite amount of solvent and spotted on the TLC plate. TLC plate was then developed using solvent system hexane: ethyl acetate (1:1 v/v). After development, TLC plate was visualized under UV-254nm, UV-365nm and derivatized with anisaldehyde reagent and ferric chloride reagent.to confirm the presence of vitexicarpin in the fractions.

*Statistical analysis*

All experiments were performed in three replicates and the results were computed as mean ± standard deviation. ANOVA (one-way) and Dunnet post-test were performed using SPSS 16.0. 

## Results

*Effects of Vitex rotundifolia extract and fractions on cytotoxicity of T-47D cell line*

**Figure 1:**
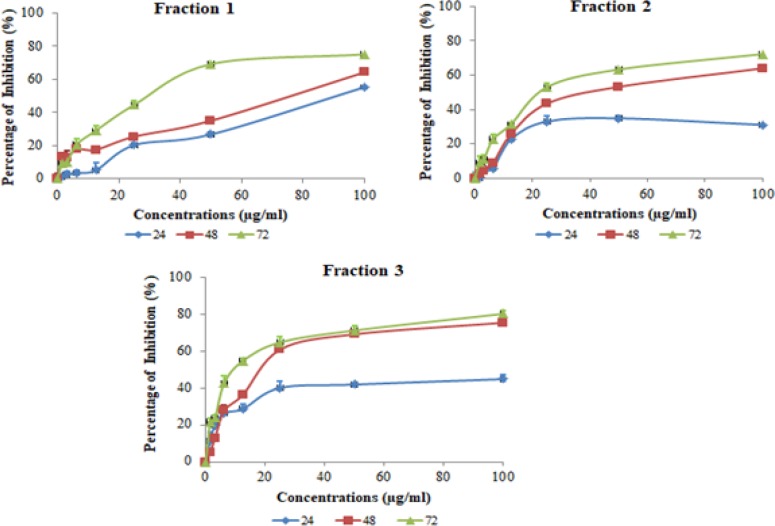
Cytotoxic Effects of Fractions of Vitex Rotundifolia on T-47D Cell Line Using MTS Assay

The crude methanol extract and seven fractions prepared from leaves of *Vitex rotundifolia* were used to investigate the cytotoxicity effects on human breast cancer cell line, T-47D by using MTS assay. The results obtained by MTS assay demonstrate that the extract and fractions of *Vitex rotundifolia* produced dose dependent inhibitory effects in T-47D cell line at all three time points used (24hr, 48hr and 72hr). Methanol extract at the concentration of 79.43µg/ml inhibited 50% growth of T-47D cells after 72hr of treatment. Out of seven fractions, F1, F2 and F3 produced cytotoxicity effects in T-47D cell line with IC_50_ (72hr) < 30µg/ml after 72hr of treatment (Figure 1). However, T-47D cell line was found to be more sensitive towards fraction F3 (dichloromethane) with IC_50_ 10µg/ml followed by F2 (dichloromethane: hexane) with IC_50 _22.38µg/ml after 72hr. Vincristine sulphate (positive control) shows the IC_50_ value of 1.25µg/ml at 72hr. The relative potential of cytotoxicity of the active fractions on T-47D cell line was found to be F3 > F2 > F1 > F5 > F4> F6 > F7.

*Vitex rotundifolia fractions induced apoptosis in T-47D cells*

**Figure 2:**
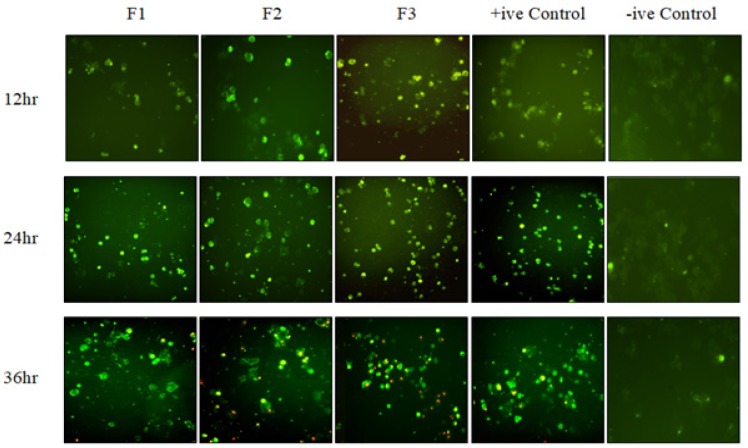
The Presence of Apoptotic Cells in the T-47D Cell Line by Annexin-V staining Assay

In order to determine the mode of cell death exhibited by the fractions in T-47D cells, annexin V-FITC apoptosis detection assay was used as an index to observe apoptotic cells. The results obtained by Annexin V-FITC staining method demonstrate that fractions F1, F2 and F3 of *Vitex rotundifolia* in T-47D cells at the concentrations of their respective IC_50_ (72hr), initiated the translocation of phosphatidylserine (PS), which shows the presence of early apoptosis in T-47D cells at 12hr and 24hr. Interestingly, at 36hr incubation, most of the cells responded positively to both Annexin V-FITC and PI which shows that late apoptosis was induced by active fractions. Thus, the results presented evidence that *Vitex rotundifolia* fractions induced cell death in T-47D cell line via apoptosis (Figure 2).

Furthermore, DeadEnd^TM^ Fluorometric Apoptosis Detection System (TUNEL assay) was performed to study DNA fragmentation (late apoptosis) induced by the active fractions of *Vitex rotundifolia* in T-47D cells. The results obtained by TUNEL assay demonstrate that the nuclei of T-47D cells were stained green when incubated with active fractions at the concentration of their respective IC_50_ (72hr) for 36hr indicating the presence of DNA fragmentation. Thus, the results strongly indicated that active fractions of *Vitex rotundifolia* induced DNA fragmentation (late apoptosis) in T-47D cell line at 36hr (Figure 2).

*Effects of Vitex rotundifolia fractions on activation of caspase and Bcl-2 family proteins in human breast cancer T-47D cells*

In order to elucidate the mechanism involved in apoptosis induced cell death in T-47D cell line, fractions F2 and F3 of *Vitex rotundifolia* were selected as cytotoxicity activity exerted by F2 and F3 was higher than F1 and F5. Western blot analysis was performed in order to examine the effect of fraction F2 and F3 of *Vitex rotundifolia* on the expressions of proteins that are responsible in inducing apoptotic cell death in T-47D cell line, caspases (Caspases - 8, 9 and 3) and Bcl-2 (Bax, Bcl-2) family proteins for 24hr. Caspase Glo^TM^ assay was performed in order to study the time-dependent activities of caspase -8, -9 and -3/7 and also to validate the results of involvement of caspases in cell death induced by the fractions of *Vitex rotundifolia* on T-47D cell line (Figure 4).

The findings obtained from western blot analysis and Caspase Glo^TM^ assay suggest that fractions F2 and F3 induced apoptotic cell death in T-47D cells may involve the activation of both caspase-8 and caspase-9. Western blot analysis demonstrate that caspase-8 was not found to be activated at 24hr in T-47D cells, however, active caspase-8 was found to be significantly increased at 36hr as determined by Caspase Glo^TM^ assay. Caspase -9 was observed to be activated at 24hr by western blot analysis which might be due to the increased expression of Pro-caspase -9 in T-47D cells treated with fractions F2 and F3 as compared to control. Caspase Glo^TM^ assay demonstrate that the activity of caspase -9 was increased significantly from 20hr to 48hr, reaching its peak at 48hr. The results obtained by western blot analysis and Caspase Glo^TM^ assay suggest the activation of effector caspases, caspase -3 and caspase -3/7 respectively, in T-47D cells treated with active fractions of *Vitex rotundifolia* indicating the involvement of caspase -3 and -7 in apoptotic cell death induced by active fractions. The activity of caspase-3/7 was increased significantly in T-47D cells from 20hr to 48hr, reaching its peak at 36hr. 

Moreover, Bcl-2 family proteins (Bax and Bcl-2) were studied by using western blot analysis. The findings indicate that the treatment of T-47D cells with fractions F2 and F3 over a period of 24hr resulted in an increase in Bax expression and decreased Bcl-2 expression or an increase in the ratio of Bax/Bcl-2 as compared to the untreated control cells.

*Identification of Vitexicarpin in active fractions of Vitex rotundifolia*

Thin layer chromatography was performed to identify the presence of standard compound Vitexicarpin (St) in the fractions of *Vitex rotundifolia* using solvent system hexane: ethyl acetate (1:1 v/v). Figure 5 shows the TLC profiling of *Vitex rotundifolia* fractions along with the standard compound visualized under UV-254 nm, UV-365 nm and after derivatization with reagents (anisaldehyde- sulphuric acid reagent and ferric chloride reagent). The identification of Vitexicarpin in fractions was done by comparing the *Rf* value of standard compound with the fractions. Vitexicarpin was observed to have a dark band excited under UV-254nm at *Rf* value of 0.32 and appeared as yellow fluorescence under UV-365nm. Spots at the same *Rf *value were also present in fractions F2, F3 and F5 when observed under UV-254nm and 365nm. After derivatization with anisaldehyde-sulphuric acid reagent and ferric chloride reagent, vitexicarpin was turned into yellow and brown colour respectively and similar spots were observed at same *Rf* value in fractions F2, F3 and F5. These findings suggest the presence of a vitexicarpin in fractions F2, F3 and F5 but absence in F1 as no spot at the standard *Rf *value was observed. 

**Figure 3:**
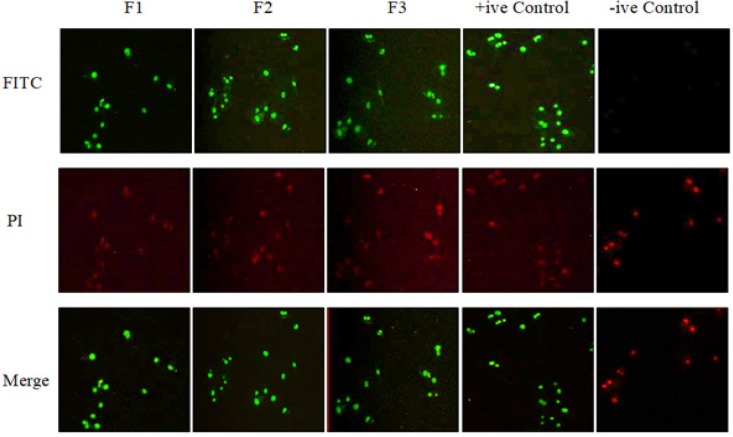
The Presence of DNA Fragmentation in Nuclei of T-47D Cells at 36 hr

**Figure 4:**
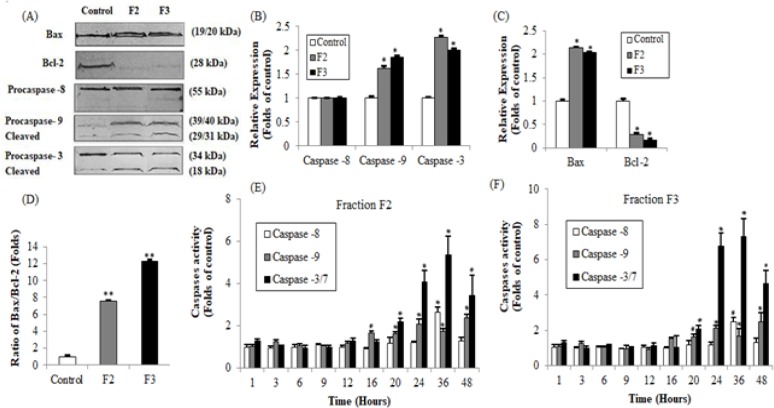
Western Blot Analysis; (A), Fraction F2 and F3 increased Bax, caspase -9, caspase -3 expression and decreased Bcl-2 expression in T-47D cells at 24hr; (B), Relative expression of Bax, Bcl-2; (C), Bax/Bcl-2 ratio in T-47D cells; (D), Relative expression of caspase -8, -9 and -3; (E), (F), Time course response of caspase-8, -9 and -3/7 activation in T-47D cells treated with fractions F2 and F3 of Vitex rotundifolia determined by Caspase GloTM assay

**Figure 5.**
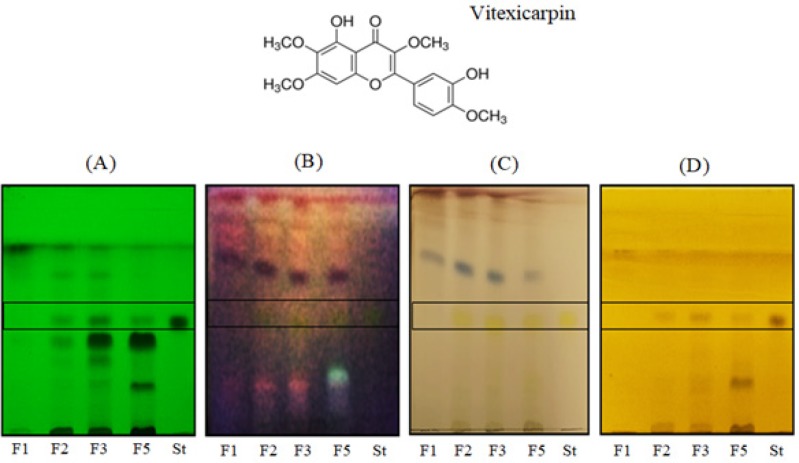
TLC Profiling of Active Fractions of Vitex Rotundifolia and Vitexicarpin (St). (A), UV-254 nm; (B), UV-254 nm; (C), anisaldehyde reagent; (D), ferric chloride reagent

**Figure 6:**
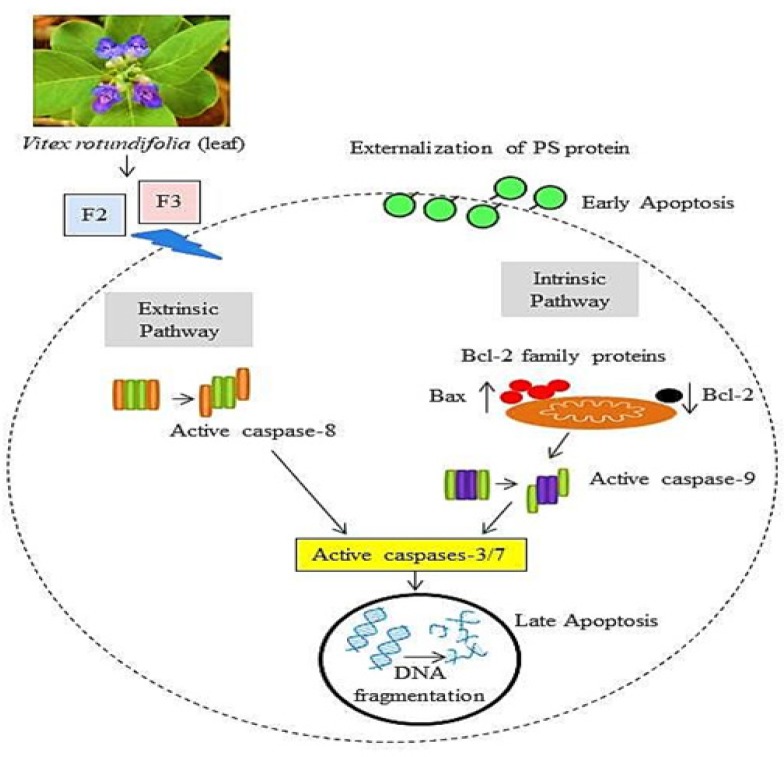
Schematic Illustration of Apoptotic Cell Death Induced by Fractions F2 and F3 of Vitex Rotundifolia in T-47D Cell Line

## Discussion

*Vitex rotundifolia* has been widely used in traditional chinese medicine (TCM) for treating inflammation and reported to have anticancer activities. The present study was carried out to investigate the cytotoxic effects and apoptosis induced by fractions of *Vitex rotundifolia* in human breast cancer cell line, T-47D. The results demonstrated that fractions of *Vitex rotundifolia* significantly reduced the growth of T-47D cells in time and dose-dependent manner. Fractions F1, F2 and F3 produced cytotoxic effects in T-47D cell line with IC_50 _< 30 µg/ml at 72hr. The relative potential of cytotoxicity of the active fractions on T-47D cell line was found to be F3 > F2 > F1. 

Similarly, previous studies that were carried out on finding the potential use of *Vitex rotundifolia* for the anticancer activity suggest that acetone extract from fruits of *Vitex rotundifolia* exhibited the cytotoxic activity on the HT-29 cells (Jo et al., 2007). Essential oil obtained from the fruits of *Vitex rotundifolia* exhibit strong estrogenic activity on proliferation of MCF-7 cells (Hu et al., 2007). Polymethoxyflavonoids namely vitexicarpin, luteolin and artemetin from fruits of *Vitex rotundifolia* were found to induce apoptosis in human myeloid leukemia cells. Rotundiuran, a diterpene isolated from the fruits of *Vitex rotundifolia* possessed apoptosis-inducing properties in human myeloid leukemia cells (Ko et al., 2000; Ko et al., 2001; Ko et al., 2002). The studies conducted to date reveal that other species of genus Vitex also possess anticancer activities against cancer cell lines. The extracts from the leaves of *Vitex negundo *and Vitex trifolia possess cytotoxicity activity in MCF-7 cell line (Arulvasu et al., 2010; Garbi et al., 2015). Organic extracts from Vitex trifolia also possess cytotoxicity activity against cervix carcinoma, ovarian and colon cancer cell lines (Hernández et al., 1999). *Vitex agnus-castus *leaf extract also induced apoptosis in hepatoma cell line HepG2 and prostate epithelial cell lines BPH-1 and PC-3 (Abdel-Lateef et al., 2016; Weisskopf et al., 2005).

The results obtained by western blot analysis and Caspase Glo^TM^ assay suggest that induction of apoptosis in T-47D cells was mediated by the activation of both caspase-8 and 9. The induction of apoptosis by the activation of caspase-9 involves intrinsic or mitochondrial pathway of apoptosis. While caspase-8 plays a major role in the extrinsic pathway of apoptosis (Loreto et al., 2014). Activation of caspase-9 and caspase-8 further activates effector cas-3, cas-6 or cas-7 (Shi, 2002; Yuan and Akey, 2013; Ouyang et al., 2012). Once activated, effector caspases induces apoptotic cell death (Wolf et al., 1999). The results obtained by western blot analysis and Caspase Glo^TM^ assay suggest the involvement of effector caspases-3/7 in apoptotic cell death induced by active fractions of *Vitex rotundifolia*. The involvement of caspase -8, -9 and -3/7 in the induction of apoptosis have been studied in plant species of genus Vitex. Casticin, a flavonoid compound from *Vitex rotundifolia* induced apoptotsis in human lung cancer cell line H157 depending on the activation of both caspase -8 and -9 (Zhou et al., 2013). Vitexin compound 1 isolated from *Vitex negundo *killed hepatocellular carcinoma cells by the activation of caspase -8, -9 and -3 (Wang et al., 2013).

Moreover, the results obtained by western blot analysis demonstrate simultaneous up-regulation of Bax and down-regulation of Bcl-2 in T-47D cell lines treated with fractions F2 and F3 at 24hr. Bax/Bcl-2 ratio was also increased in fractions treated T-47D cell line as compared to untreated control cells. Bcl-2 family proteins play a vital role in regulating mitochondrial mediated apoptotic pathway (Hardwick et al., 2013). Pro-apoptotic member of Bcl-2 family proteins such as Bax promotes apoptosis while the anti-apoptotic proteins of Bcl-2 family such as Bcl-2 restrain apoptosis. The ratio of pro-apoptotic and anti-apoptotic proteins determines the fate of the cell (An et al., 2004). Up-regulation of Bax and down-regulation of Bcl-2 is usually associated with the cell death and by using the ratio of Bax / Bcl-2 proteins, the fate of cell could be determined (Naseri et al., 2015; Gul-e-Saba et al., 2019). These findings were in parallel with previous studies performed on *Vitex rotundifolia* and other species of genus Vitex. Casticin isolated from *Vitex rotundifolia* induced apoptotsis in human gall bladder cancer cell lines SGC996 and NOZ by upregulation of Bax and down-regulation of Bcl-2 protein (Song et al., 2017). Lignans compound such as Vitexins isolated from seeds of *Vitex Negundo *induced apoptosis in breast cancer, ovarian cancer and prostate cancer cell lines by up-regulating Bax and down-regulating Bcl-2 protein (Zhou et al., 2009).

Furthermore, TLC analysis confirms the presence of vitexicarpin in active fractions of *Vitex rotundifolia*. Vitexicarpin have been previously reported to possess anticancer activities (Wang et al., 2005; Zhou et al., 2013; Song et al., 2017), therefore, the presence of vitexicarpin in the fractions might result in their cytotoxicity activity. 

These findings suggest the involvement of caspase -8 and mitochondrial pathway of apoptosis in cell death induced by active fractions of *Vitex rotundifolia* in T-47D cell line. The schematic illustration of apoptotic cell death induced by fractions F2 and F3 of *Vitex rotundifolia* in T-47D cell line is shown in Figure 6.

In conclusion, the present study shows that the fractions prepared from leaves of *Vitex rotundifolia* produced potent cytotoxicity against breast cancer cells, T47D. The mode of cell death depicts apoptosis as a major mode of cell death via both intrinsic and extrinsic apoptotic pathway. Moreover, chemical profiling confirms the fractions of *Vitex rotundifolia* possess potential active metabolites of future cancer therapeutics. 
